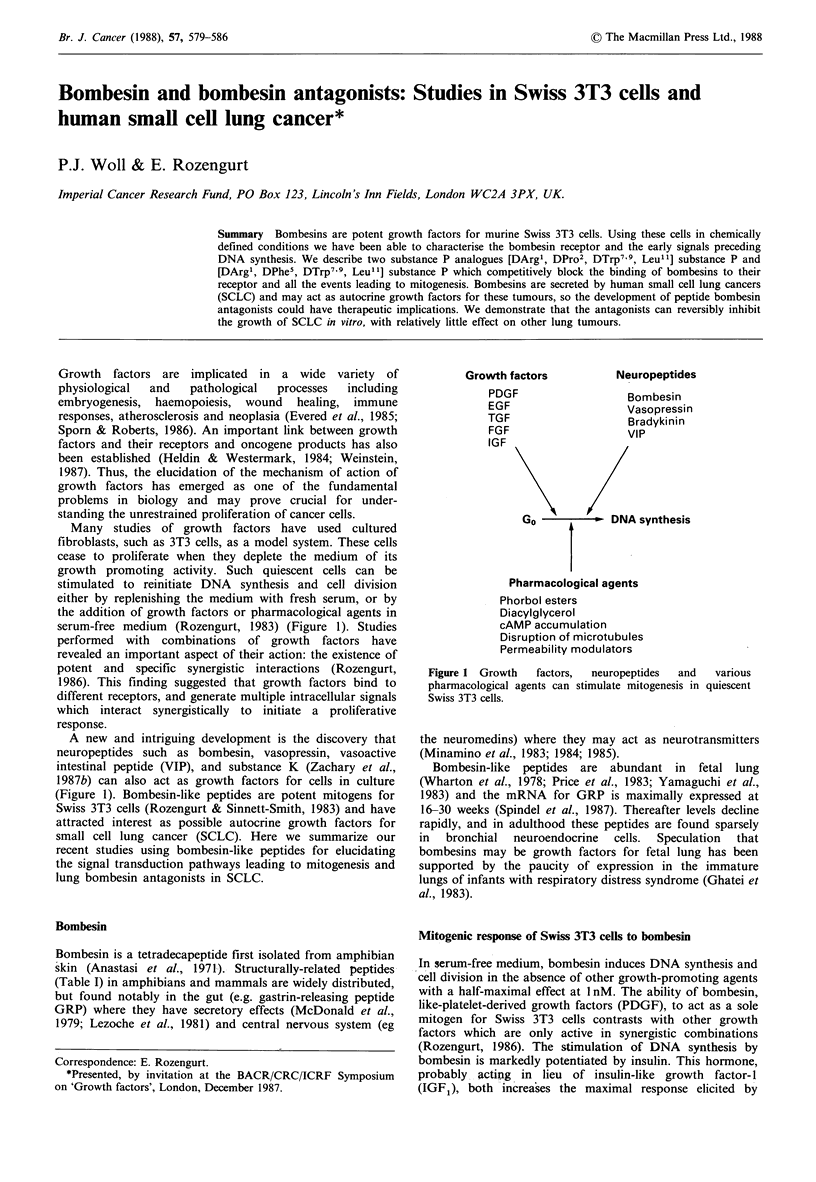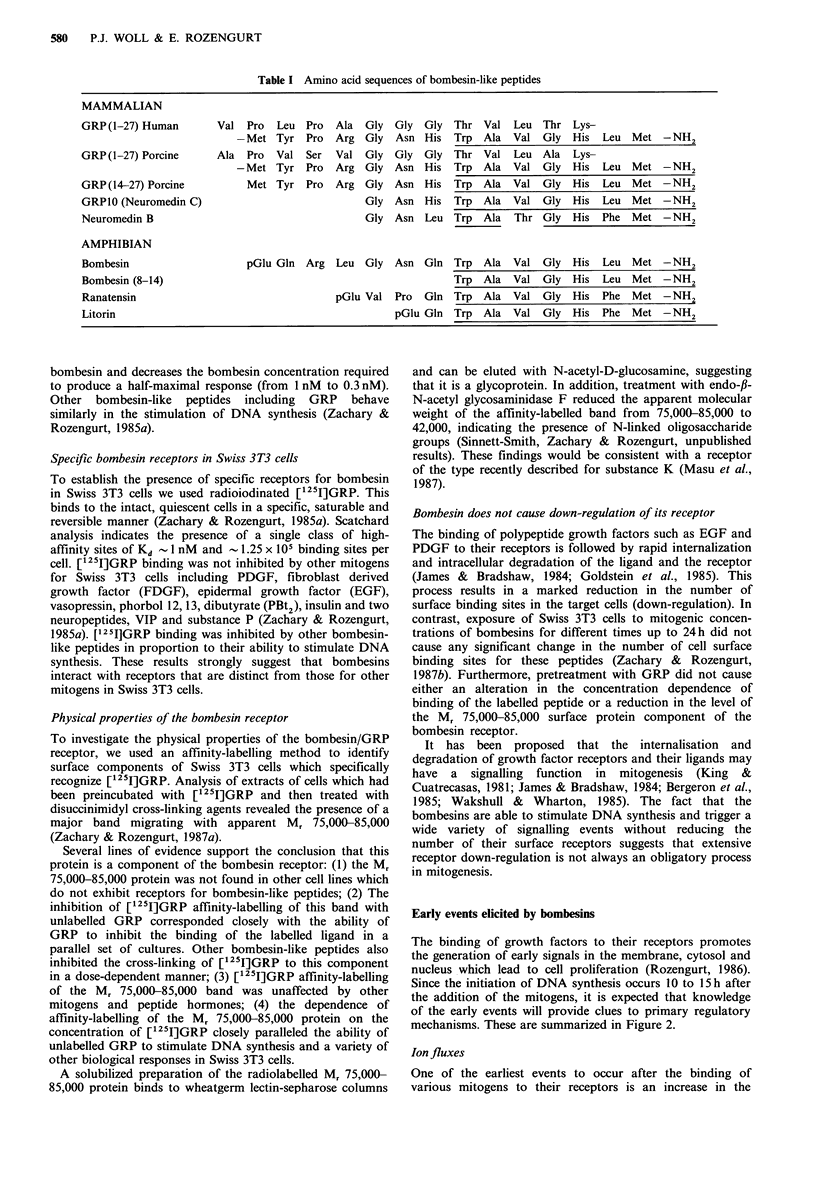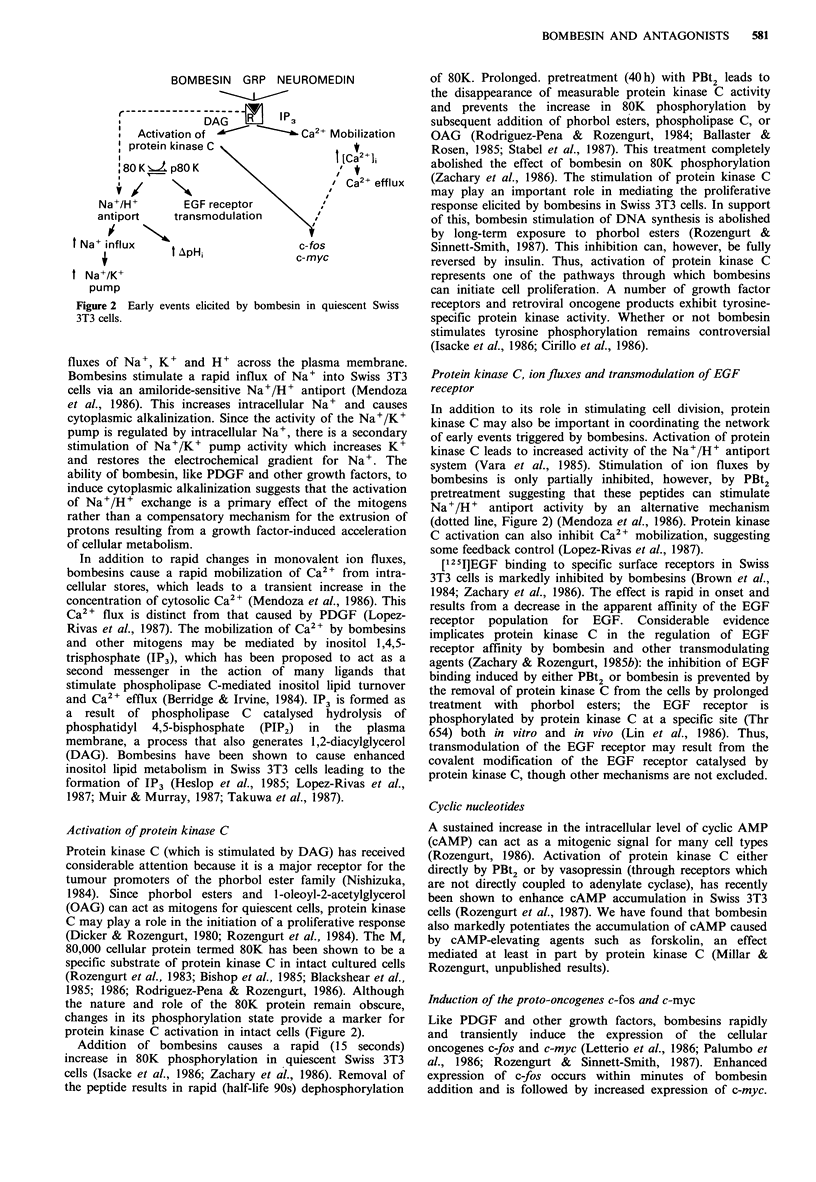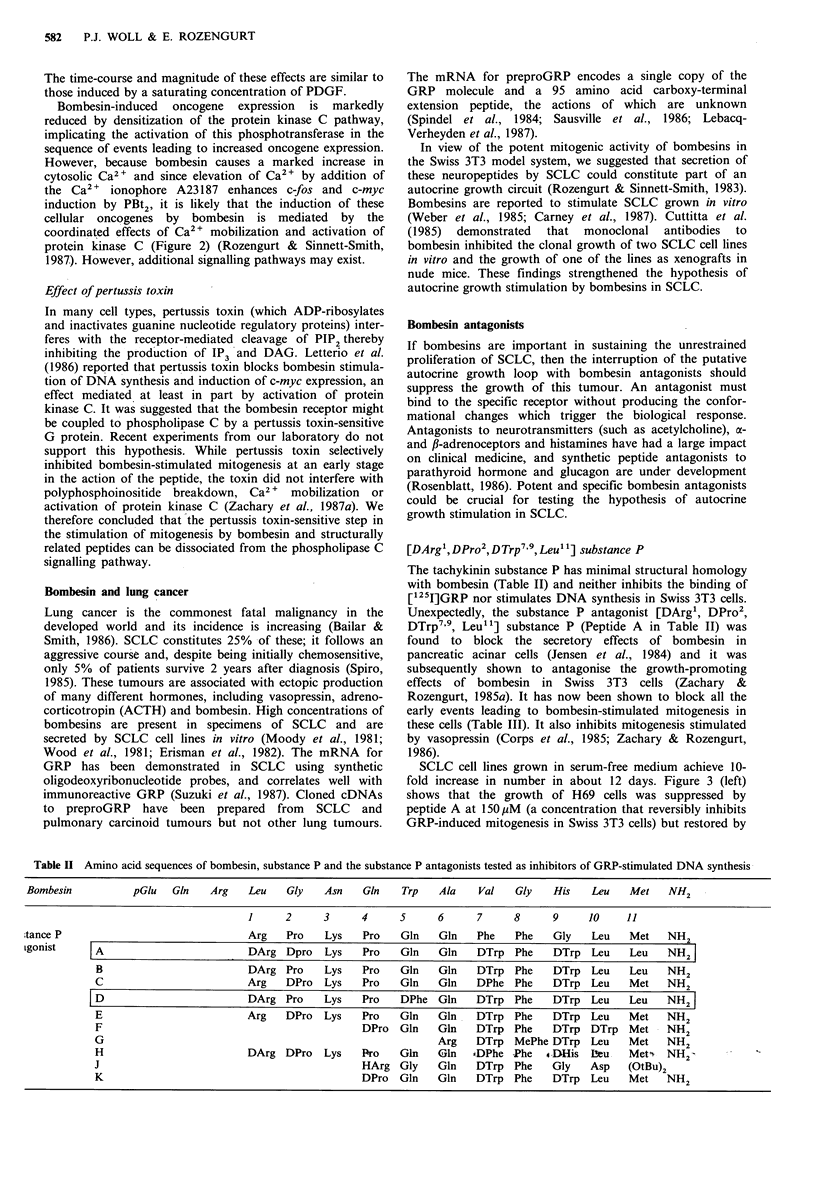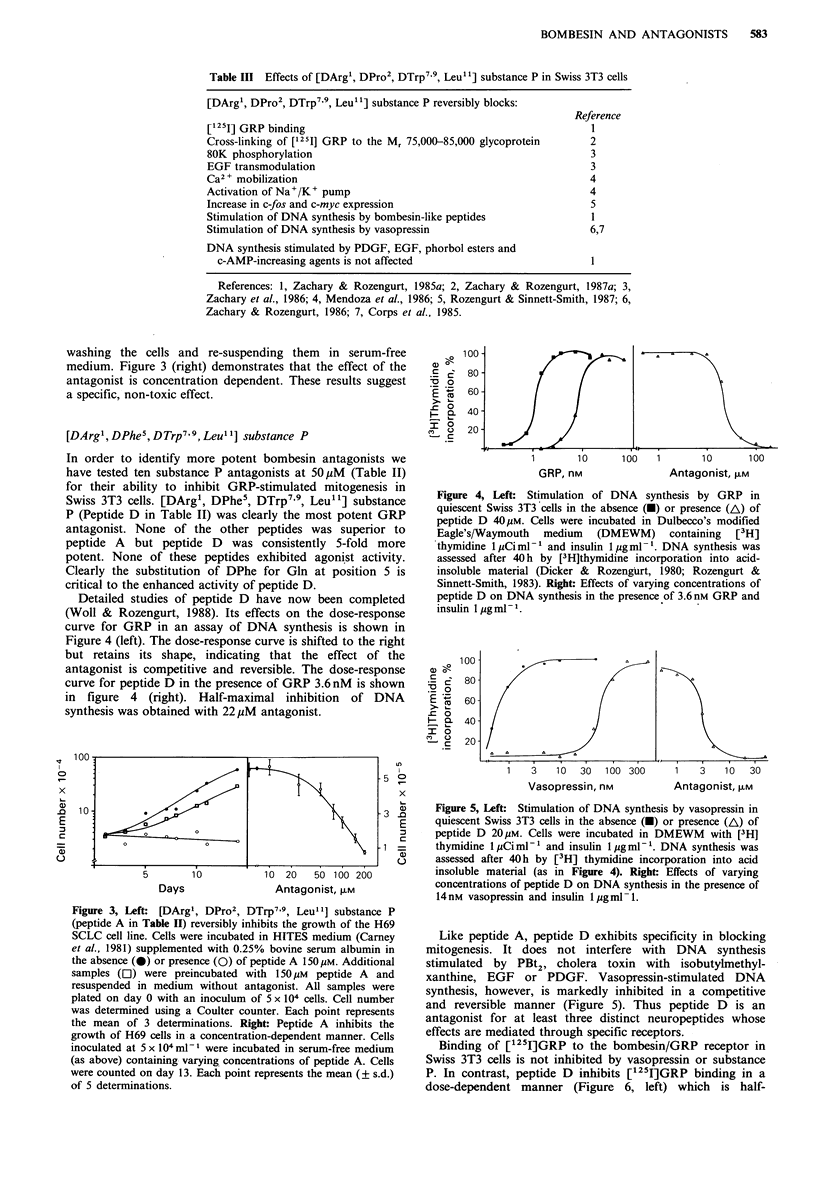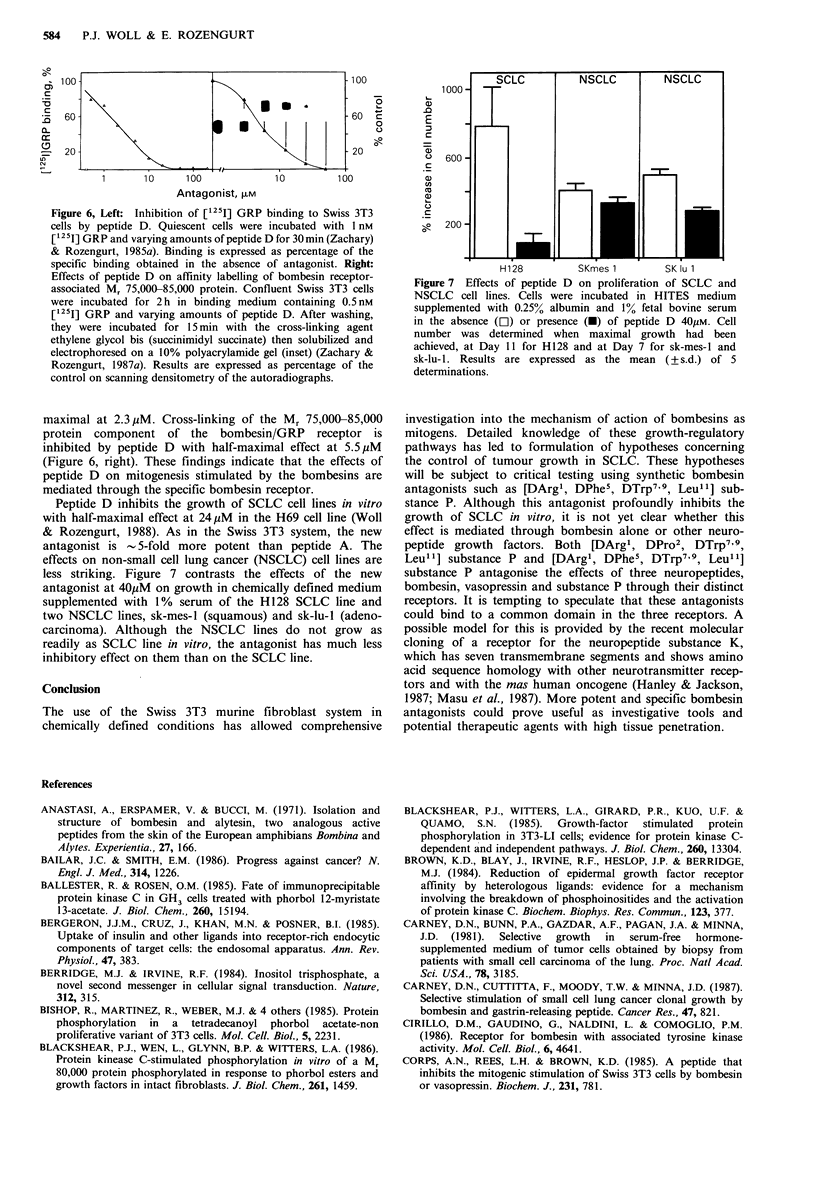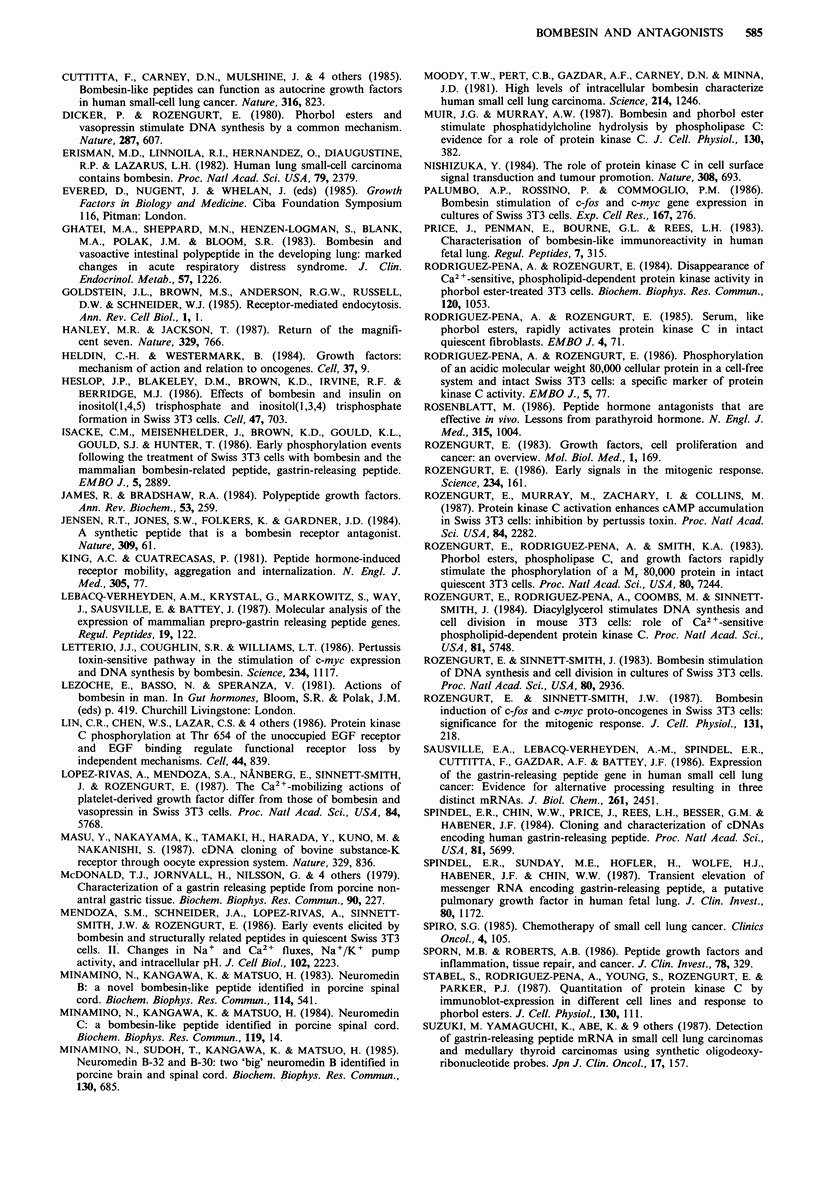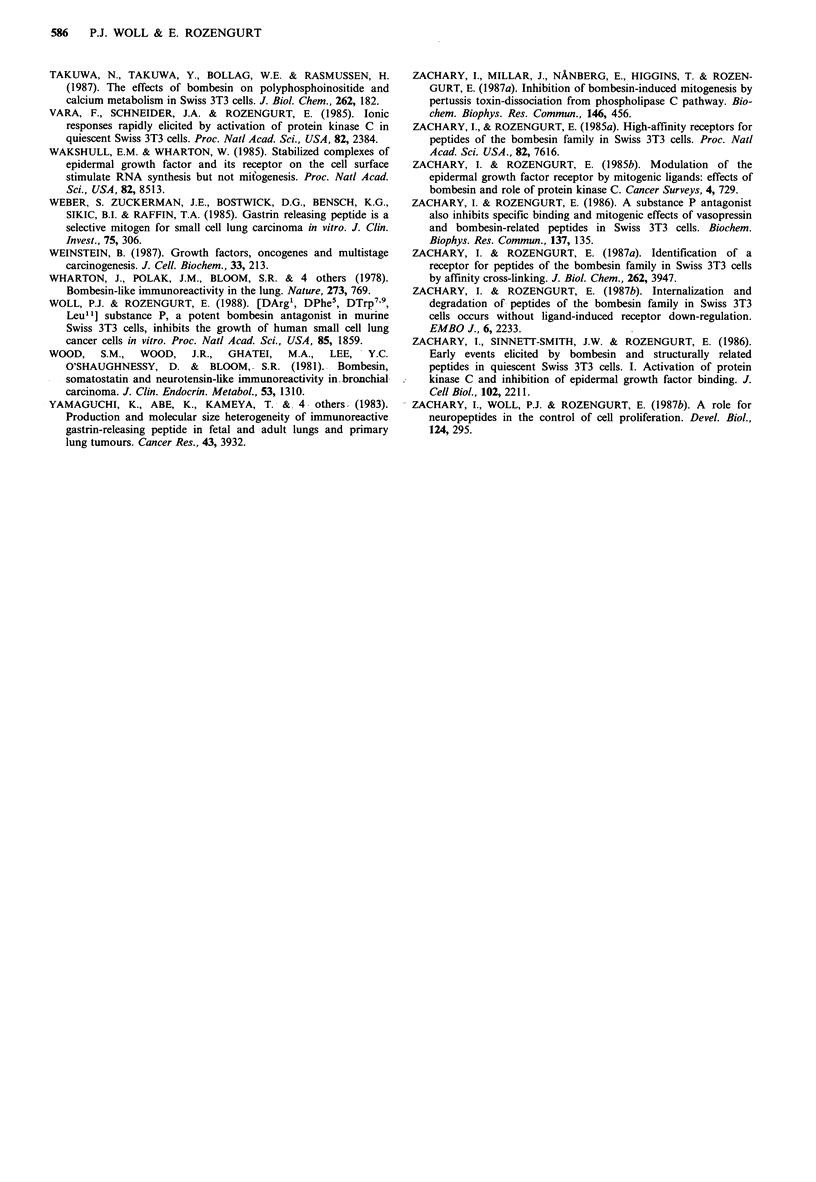# Bombesin and bombesin antagonists: studies in Swiss 3T3 cells and human small cell lung cancer.

**DOI:** 10.1038/bjc.1988.132

**Published:** 1988-06

**Authors:** P. J. Woll, E. Rozengurt

**Affiliations:** Imperial Cancer Research Fund, Lincoln's Inn Fields, London, UK.

## Abstract

Bombesins are potent growth factors for murine Swiss 3T3 cells. Using these cells in chemically defined conditions we have been able to characterise the bombesin receptor and the early signals preceding DNA synthesis. We describe two substance P analogues [DArg1, DPro2, DTrp7,9, Leu11] substance P and [DArg1, DPhe5, DTrp7,9, Leu11] substance P which competitively block the binding of bombesins to their receptor and all the events leading to mitogenesis. Bombesins are secreted by human small cell lung cancers (SCLC) and may act as autocrine growth factors for these tumours, so the development of peptide bombesin antagonists could have therapeutic implications. We demonstrate that the antagonists can reversibly inhibit the growth of SCLC in vitro, with relatively little effect on other lung tumours.


*Br. J. Cancer* (1988), **57**, 579–586 © The Macmillan Press Ltd., 1988

### Bombesin and bombesin antagonists: Studies in Swiss 3T3 cells and human small cell lung cancer*

P.J. Woll & E. Rozengurt

*Imperial Cancer Research Fund, PO Box 123, Lincoln's Inn Fields, London WC2A 3PX, UK.*

**Summary** Bombesins are potent growth factors for murine Swiss 3T3 cells. Using these cells in chemically defined conditions we have been able to characterise the bombesin receptor and the early signals preceding DNA synthesis. We describe two substance P analogues [DArg$^1$, DPro$^2$, DTrp$^{7,9}$, Leu$^{11}$] substance P and [DArg$^1$, DPhe$^5$, DTrp$^{7,9}$, Leu$^{11}$] substance P which competitively block the binding of bombesins to their receptor and all the events leading to mitogenesis. Bombesins are secreted by human small cell lung cancers (SCLC) and may act as autocrine growth factors for these tumours, so the development of peptide bombesin antagonists could have therapeutic implications. We demonstrate that the antagonists can reversibly inhibit the growth of SCLC *in vitro*, with relatively little effect on other lung tumours.

Growth factors are implicated in a wide variety of physiological and pathological processes including embryogenesis, haemopoiesis, wound healing, immune responses, atherosclerosis and neoplasia (Evered *et al*., 1985; Sporn & Roberts, 1986). An important link between growth factors and their receptors and oncogene products has also been established (Heldin & Westermark, 1984; Weinstein, 1987). Thus, the elucidation of the mechanism of action of growth factors has emerged as one of the fundamental problems in biology and may prove crucial for understanding the unrestrained proliferation of cancer cells.

Many studies of growth factors have used cultured fibroblasts, such as 3T3 cells, as a model system. These cells cease to proliferate when they deplete the medium of its growth promoting activity. Such quiescent cells can be stimulated to reinitiate DNA synthesis and cell division either by replenishing the medium with fresh serum, or by the addition of growth factors or pharmacological agents in serum-free medium (Rozengurt, 1983) (Figure 1). Studies performed with combinations of growth factors have revealed an important aspect of their action: the existence of potent and specific synergistic interactions (Rozengurt, 1986). This finding suggested that growth factors bind to different receptors, and generate multiple intracellular signals which interact synergistically to initiate a proliferative response.

A new and intriguing development is the discovery that neuropeptides such as bombesin, vasopressin, vasoactive intestinal peptide (VIP), and substance K (Zachary *et al*., 1987*b*) can also act as growth factors for cells in culture (Figure 1). Bombesin-like peptides are potent mitogens for Swiss 3T3 cells (Rozengurt & Sinnett-Smith, 1983) and have attracted interest as possible autocrine growth factors for small cell lung cancer (SCLC). Here we summarize our recent studies using bombesin-like peptides for elucidating the signal transduction pathways leading to mitogenesis and lung bombesin antagonists in SCLC.

**Figure 1** Growth factors, neuropeptides and various pharmacological agents can stimulate mitogenesis in quiescent Swiss 3T3 cells.

### Bombesin

Bombesin is a tetradecapeptide first isolated from amphibian skin (Anastasi *et al*., 1971). Structurally-related peptides (Table I) in amphibians and mammals are widely distributed, but found notably in the gut (e.g. gastrin-releasing peptide GRP) where they have secretory effects (McDonald *et al*., 1979; Lezoche *et al*., 1981) and central nervous system (eg the neuromedins) where they may act as neurotransmitters (Minamino *et al*., 1983; 1984; 1985).

Bombesin-like peptides are abundant in fetal lung (Wharton *et al*., 1978; Price *et al*., 1983; Yamaguchi *et al*., 1983) and the mRNA for GRP is maximally expressed at 16–30 weeks (Spindel *et al*., 1987). Thereafter levels decline rapidly, and in adulthood these peptides are found sparsely in bronchial neuroendocrine cells. Speculation that bombesins may be growth factors for fetal lung has been supported by the paucity of expression in the immature lungs of infants with respiratory distress syndrome (Ghatei *et al*., 1983).

### Mitogenic response of Swiss 3T3 cells to bombesin

In serum-free medium, bombesin induces DNA synthesis and cell division in the absence of other growth-promoting agents with a half-maximal effect at 1 nM. The ability of bombesin, like-platelet-derived growth factors (PDGF), to act as a sole mitogen for Swiss 3T3 cells contrasts with other growth factors which are only active in synergistic combinations (Rozengurt, 1986). The stimulation of DNA synthesis by bombesin is markedly potentiated by insulin. This hormone, probably acting in lieu of insulin-like growth factor-1 ($IGF_1$), both increases the maximal response elicited by

Correspondence: E. Rozengurt.

*Presented, by invitation at the BACR/CRC/ICRF Symposium on 'Growth factors', London, December 1987.

**Table I** Amino acid sequences of bombesin-like peptides

| | | | | | | | | | | | | | | | | |
|---|---|---|---|---|---|---|---|---|---|---|---|---|---|---|---|---|
| MAMMALIAN | | | | | | | | | | | | | | | | |
| GRP(1–27) Human | Val | Pro | Leu | Pro | Ala | Gly | Gly | Gly | Thr | Val | Leu | Thr | Lys– | | | |
| | | –Met | Tyr | Pro | Arg | Gly | Asn | His | Trp | Ala | Val | Gly | His | Leu | Met | $-NH_2$ |
| GRP(1–27) Porcine | Ala | Pro | Val | Ser | Val | Gly | Gly | Gly | Thr | Val | Leu | Ala | Lys– | | | |
| | | –Met | Tyr | Pro | Arg | Gly | Asn | His | Trp | Ala | Val | Gly | His | Leu | Met | $-NH_2$ |
| GRP(14–27) Porcine | | Met | Tyr | Pro | Arg | Gly | Asn | His | Trp | Ala | Val | Gly | His | Leu | Met | $-NH_2$ |
| GRP10 (Neuromedin C) | | | | | | Gly | Asn | His | Trp | Ala | Val | Gly | His | Leu | Met | $-NH_2$ |
| Neuromedin B | | | | | | Gly | Asn | Leu | Trp | Ala | Thr | Gly | His | Phe | Met | $-NH_2$ |
| AMPHIBIAN | | | | | | | | | | | | | | | | |
| Bombesin | | pGlu | Gln | Arg | Leu | Gly | Asn | Gln | Trp | Ala | Val | Gly | His | Leu | Met | $-NH_2$ |
| Bombesin (8–14) | | | | | | | | | Trp | Ala | Val | Gly | His | Leu | Met | $-NH_2$ |
| Ranatensin | | | | | pGlu | Val | Pro | Gln | Trp | Ala | Val | Gly | His | Phe | Met | $-NH_2$ |
| Litorin | | | | | | | pGlu | Gln | Trp | Ala | Val | Gly | His | Phe | Met | $-NH_2$ |

bombesin and decreases the bombesin concentration required to produce a half-maximal response (from 1 nM to 0.3 nM). Other bombesin-like peptides including GRP behave similarly in the stimulation of DNA synthesis (Zachary & Rozengurt, 1985*a*).

*Specific bombesin receptors in Swiss 3T3 cells*

To establish the presence of specific receptors for bombesin in Swiss 3T3 cells we used radioiodinated [$^{125}$I]GRP. This binds to the intact, quiescent cells in a specific, saturable and reversible manner (Zachary & Rozengurt, 1985*a*). Scatchard analysis indicates the presence of a single class of high-affinity sites of $K_d$ ~1 nM and ~$1.25 \times 10^5$ binding sites per cell. [$^{125}$I]GRP binding was not inhibited by other mitogens for Swiss 3T3 cells including PDGF, fibroblast derived growth factor (FDGF), epidermal growth factor (EGF), vasopressin, phorbol 12, 13, dibutyrate ($PBt_2$), insulin and two neuropeptides, VIP and substance P (Zachary & Rozengurt, 1985*a*). [$^{125}$I]GRP binding was inhibited by other bombesin-like peptides in proportion to their ability to stimulate DNA synthesis. These results strongly suggest that bombesins interact with receptors that are distinct from those for other mitogens in Swiss 3T3 cells.

*Physical properties of the bombesin receptor*

To investigate the physical properties of the bombesin/GRP receptor, we used an affinity-labelling method to identify surface components of Swiss 3T3 cells which specifically recognize [$^{125}$I]GRP. Analysis of extracts of cells which had been preincubated with [$^{125}$I]GRP and then treated with disuccinimidyl cross-linking agents revealed the presence of a major band migrating with apparent $M_r$ 75,000–85,000 (Zachary & Rozengurt, 1987*a*).

Several lines of evidence support the conclusion that this protein is a component of the bombesin receptor: (1) the $M_r$ 75,000–85,000 protein was not found in other cell lines which do not exhibit receptors for bombesin-like peptides; (2) The inhibition of [$^{125}$I]GRP affinity-labelling of this band with unlabelled GRP corresponded closely with the ability of GRP to inhibit the binding of the labelled ligand in a parallel set of cultures. Other bombesin-like peptides also inhibited the cross-linking of [$^{125}$I]GRP to this component in a dose-dependent manner; (3) [$^{125}$I]GRP affinity-labelling of the $M_r$ 75,000–85,000 band was unaffected by other mitogens and peptide hormones; (4) the dependence of affinity-labelling of the $M_r$ 75,000–85,000 protein on the concentration of [$^{125}$I]GRP closely paralleled the ability of unlabelled GRP to stimulate DNA synthesis and a variety of other biological responses in Swiss 3T3 cells.

A solubilized preparation of the radiolabelled $M_r$ 75,000–85,000 protein binds to wheatgerm lectin-sepharose columns and can be eluted with N-acetyl-D-glucosamine, suggesting that it is a glycoprotein. In addition, treatment with endo-$\beta$-N-acetyl glycosaminidase F reduced the apparent molecular weight of the affinity-labelled band from 75,000–85,000 to 42,000, indicating the presence of N-linked oligosaccharide groups (Sinnett-Smith, Zachary & Rozengurt, unpublished results). These findings would be consistent with a receptor of the type recently described for substance K (Masu *et al.*, 1987).

*Bombesin does not cause down-regulation of its receptor*

The binding of polypeptide growth factors such as EGF and PDGF to their receptors is followed by rapid internalization and intracellular degradation of the ligand and the receptor (James & Bradshaw, 1984; Goldstein *et al.*, 1985). This process results in a marked reduction in the number of surface binding sites in the target cells (down-regulation). In contrast, exposure of Swiss 3T3 cells to mitogenic concentrations of bombesins for different times up to 24 h did not cause any significant change in the number of cell surface binding sites for these peptides (Zachary & Rozengurt, 1987*b*). Furthermore, pretreatment with GRP did not cause either an alteration in the concentration dependence of binding of the labelled peptide or a reduction in the level of the $M_r$ 75,000–85,000 surface protein component of the bombesin receptor.

It has been proposed that the internalisation and degradation of growth factor receptors and their ligands may have a signalling function in mitogenesis (King & Cuatrecasas, 1981; James & Bradshaw, 1984; Bergeron *et al.*, 1985; Wakshull & Wharton, 1985). The fact that the bombesins are able to stimulate DNA synthesis and trigger a wide variety of signalling events without reducing the number of their surface receptors suggests that extensive receptor down-regulation is not always an obligatory process in mitogenesis.

### Early events elicited by bombesins

The binding of growth factors to their receptors promotes the generation of early signals in the membrane, cytosol and nucleus which lead to cell proliferation (Rozengurt, 1986). Since the initiation of DNA synthesis occurs 10 to 15 h after the addition of the mitogens, it is expected that knowledge of the early events will provide clues to primary regulatory mechanisms. These are summarized in Figure 2.

*Ion fluxes*

One of the earliest events to occur after the binding of various mitogens to their receptors is an increase in the

**Figure 2** Early events elicited by bombesin in quiescent Swiss 3T3 cells.

fluxes of $Na^+$, $K^+$ and $H^+$ across the plasma membrane. Bombesins stimulate a rapid influx of $Na^+$ into Swiss 3T3 cells via an amiloride-sensitive $Na^+/H^+$ antiport (Mendoza *et al.*, 1986). This increases intracellular $Na^+$ and causes cytoplasmic alkalinization. Since the activity of the $Na^+/K^+$ pump is regulated by intracellular $Na^+$, there is a secondary stimulation of $Na^+/K^+$ pump activity which increases $K^+$ and restores the electrochemical gradient for $Na^+$. The ability of bombesin, like PDGF and other growth factors, to induce cytoplasmic alkalinization suggests that the activation of $Na^+/H^+$ exchange is a primary effect of the mitogens rather than a compensatory mechanism for the extrusion of protons resulting from a growth factor-induced acceleration of cellular metabolism.

In addition to rapid changes in monovalent ion fluxes, bombesins cause a rapid mobilization of $Ca^{2+}$ from intracellular stores, which leads to a transient increase in the concentration of cytosolic $Ca^{2+}$ (Mendoza *et al.*, 1986). This $Ca^{2+}$ flux is distinct from that caused by PDGF (Lopez-Rivas *et al.*, 1987). The mobilization of $Ca^{2+}$ by bombesins and other mitogens may be mediated by inositol 1,4,5-trisphosphate ($IP_3$), which has been proposed to act as a second messenger in the action of many ligands that stimulate phospholipase C-mediated inositol lipid turnover and $Ca^{2+}$ efflux (Berridge & Irvine, 1984). $IP_3$ is formed as a result of phospholipase C catalysed hydrolysis of phosphatidyl 4,5-bisphosphate ($PIP_2$) in the plasma membrane, a process that also generates 1,2-diacylglycerol (DAG). Bombesins have been shown to cause enhanced inositol lipid metabolism in Swiss 3T3 cells leading to the formation of $IP_3$ (Heslop *et al.*, 1985; Lopez-Rivas *et al.*, 1987; Muir & Murray, 1987; Takuwa *et al.*, 1987).

### *Activation of protein kinase C*

Protein kinase C (which is stimulated by DAG) has received considerable attention because it is a major receptor for the tumour promoters of the phorbol ester family (Nishizuka, 1984). Since phorbol esters and 1-oleoyl-2-acetylglycerol (OAG) can act as mitogens for quiescent cells, protein kinase C may play a role in the initiation of a proliferative response (Dicker & Rozengurt, 1980; Rozengurt *et al.*, 1984). The $M_r$ 80,000 cellular protein termed 80K has been shown to be a specific substrate of protein kinase C in intact cultured cells (Rozengurt *et al.*, 1983; Bishop *et al.*, 1985; Blackshear *et al.*, 1985; 1986; Rodriguez-Pena & Rozengurt, 1986). Although the nature and role of the 80K protein remain obscure, changes in its phosphorylation state provide a marker for protein kinase C activation in intact cells (Figure 2).

Addition of bombesins causes a rapid (15 seconds) increase in 80K phosphorylation in quiescent Swiss 3T3 cells (Isacke *et al.*, 1986; Zachary *et al.*, 1986). Removal of the peptide results in rapid (half-life 90s) dephosphorylation of 80K. Prolonged. pretreatment (40 h) with $PBt_2$ leads to the disappearance of measurable protein kinase C activity and prevents the increase in 80K phosphorylation by subsequent addition of phorbol esters, phospholipase C, or OAG (Rodriguez-Pena & Rozengurt, 1984; Ballaster & Rosen, 1985; Stabel *et al.*, 1987). This treatment completely abolished the effect of bombesin on 80K phosphorylation (Zachary *et al.*, 1986). The stimulation of protein kinase C may play an important role in mediating the proliferative response elicited by bombesins in Swiss 3T3 cells. In support of this, bombesin stimulation of DNA synthesis is abolished by long-term exposure to phorbol esters (Rozengurt & Sinnett-Smith, 1987). This inhibition can, however, be fully reversed by insulin. Thus, activation of protein kinase C represents one of the pathways through which bombesins can initiate cell proliferation. A number of growth factor receptors and retroviral oncogene products exhibit tyrosine-specific protein kinase activity. Whether or not bombesin stimulates tyrosine phosphorylation remains controversial (Isacke *et al.*, 1986; Cirillo *et al.*, 1986).

### *Protein kinase C, ion fluxes and transmodulation of EGF receptor*

In addition to its role in stimulating cell division, protein kinase C may also be important in coordinating the network of early events triggered by bombesins. Activation of protein kinase C leads to increased activity of the $Na^+/H^+$ antiport system (Vara *et al.*, 1985). Stimulation of ion fluxes by bombesins is only partially inhibited, however, by $PBt_2$ pretreatment suggesting that these peptides can stimulate $Na^+/H^+$ antiport activity by an alternative mechanism (dotted line, Figure 2) (Mendoza *et al.*, 1986). Protein kinase C activation can also inhibit $Ca^{2+}$ mobilization, suggesting some feedback control (Lopez-Rivas *et al.*, 1987).

[$^{125}$I]EGF binding to specific surface receptors in Swiss 3T3 cells is markedly inhibited by bombesins (Brown *et al.*, 1984; Zachary *et al.*, 1986). The effect is rapid in onset and results from a decrease in the apparent affinity of the EGF receptor population for EGF. Considerable evidence implicates protein kinase C in the regulation of EGF receptor affinity by bombesin and other transmodulating agents (Zachary & Rozengurt, 1985*b*): the inhibition of EGF binding induced by either $PBt_2$ or bombesin is prevented by the removal of protein kinase C from the cells by prolonged treatment with phorbol esters; the EGF receptor is phosphorylated by protein kinase C at a specific site (Thr 654) both *in vitro* and *in vivo* (Lin *et al.*, 1986). Thus, transmodulation of the EGF receptor may result from the covalent modification of the EGF receptor catalysed by protein kinase C, though other mechanisms are not excluded.

### *Cyclic nucleotides*

A sustained increase in the intracellular level of cyclic AMP (cAMP) can act as a mitogenic signal for many cell types (Rozengurt, 1986). Activation of protein kinase C either directly by $PBt_2$ or by vasopressin (through receptors which are not directly coupled to adenylate cyclase), has recently been shown to enhance cAMP accumulation in Swiss 3T3 cells (Rozengurt *et al.*, 1987). We have found that bombesin also markedly potentiates the accumulation of cAMP caused by cAMP-elevating agents such as forskolin, an effect mediated at least in part by protein kinase C (Millar & Rozengurt, unpublished results).

### *Induction of the proto-oncogenes c-*fos *and c-*myc

Like PDGF and other growth factors, bombesins rapidly and transiently induce the expression of the cellular oncogenes c-*fos* and c-*myc* (Letterio *et al.*, 1986; Palumbo *et al.*, 1986; Rozengurt & Sinnett-Smith, 1987). Enhanced expression of c-*fos* occurs within minutes of bombesin addition and is followed by increased expression of c-*myc*.

The time-course and magnitude of these effects are similar to those induced by a saturating concentration of PDGF.

Bombesin-induced oncogene expression is markedly reduced by densitization of the protein kinase C pathway, implicating the activation of this phosphotransferase in the sequence of events leading to increased oncogene expression. However, because bombesin causes a marked increase in cytosolic $Ca^{2+}$ and since elevation of $Ca^{2+}$ by addition of the $Ca^{2+}$ ionophore A23187 enhances c-*fos* and c-*myc* induction by $PBt_2$, it is likely that the induction of these cellular oncogenes by bombesin is mediated by the coordinated effects of $Ca^{2+}$ mobilization and activation of protein kinase C (Figure 2) (Rozengurt & Sinnett-Smith, 1987). However, additional signalling pathways may exist.

*Effect of pertussis toxin*

In many cell types, pertussis toxin (which ADP-ribosylates and inactivates guanine nucleotide regulatory proteins) interferes with the receptor-mediated cleavage of $PIP_2$ thereby inhibiting the production of $IP_3$ and DAG. Letterio *et al.* (1986) reported that pertussis toxin blocks bombesin stimulation of DNA synthesis and induction of c-*myc* expression, an effect mediated at least in part by activation of protein kinase C. It was suggested that the bombesin receptor might be coupled to phospholipase C by a pertussis toxin-sensitive G protein. Recent experiments from our laboratory do not support this hypothesis. While pertussis toxin selectively inhibited bombesin-stimulated mitogenesis at an early stage in the action of the peptide, the toxin did not interfere with polyphosphoinositide breakdown, $Ca^{2+}$ mobilization or activation of protein kinase C (Zachary *et al.*, 1987*a*). We therefore concluded that the pertussis toxin-sensitive step in the stimulation of mitogenesis by bombesin and structurally related peptides can be dissociated from the phospholipase C signalling pathway.

### Bombesin and lung cancer

Lung cancer is the commonest fatal malignancy in the developed world and its incidence is increasing (Bailar & Smith, 1986). SCLC constitutes 25% of these; it follows an aggressive course and, despite being initially chemosensitive, only 5% of patients survive 2 years after diagnosis (Spiro, 1985). These tumours are associated with ectopic production of many different hormones, including vasopressin, adrenocorticotropin (ACTH) and bombesin. High concentrations of bombesins are present in specimens of SCLC and are secreted by SCLC cell lines *in vitro* (Moody *et al.*, 1981; Wood *et al.*, 1981; Erisman *et al.*, 1982). The mRNA for GRP has been demonstrated in SCLC using synthetic oligodeoxyribonucleotide probes, and correlates well with immunoreactive GRP (Suzuki *et al.*, 1987). Cloned cDNAs to preproGRP have been prepared from SCLC and pulmonary carcinoid tumours but not other lung tumours. The mRNA for preproGRP encodes a single copy of the GRP molecule and a 95 amino acid carboxy-terminal extension peptide, the actions of which are unknown (Spindel *et al.*, 1984; Sausville *et al.*, 1986; Lebacq-Verheyden *et al.*, 1987).

In view of the potent mitogenic activity of bombesins in the Swiss 3T3 model system, we suggested that secretion of these neuropeptides by SCLC could constitute part of an autocrine growth circuit (Rozengurt & Sinnett-Smith, 1983). Bombesins are reported to stimulate SCLC grown *in vitro* (Weber *et al.*, 1985; Carney *et al.*, 1987). Cuttitta *et al.* (1985) demonstrated that monoclonal antibodies to bombesin inhibited the clonal growth of two SCLC cell lines *in vitro* and the growth of one of the lines as xenografts in nude mice. These findings strengthened the hypothesis of autocrine growth stimulation by bombesins in SCLC.

### Bombesin antagonists

If bombesins are important in sustaining the unrestrained proliferation of SCLC, then the interruption of the putative autocrine growth loop with bombesin antagonists should suppress the growth of this tumour. An antagonist must bind to the specific receptor without producing the conformational changes which trigger the biological response. Antagonists to neurotransmitters (such as acetylcholine), α- and β-adrenoceptors and histamines have had a large impact on clinical medicine, and synthetic peptide antagonists to parathyroid hormone and glucagon are under development (Rosenblatt, 1986). Potent and specific bombesin antagonists could be crucial for testing the hypothesis of autocrine growth stimulation in SCLC.

*[DArg$^1$,DPro$^2$,DTrp$^{7,9}$,Leu$^{11}$] substance P*

The tachykinin substance P has minimal structural homology with bombesin (Table II) and neither inhibits the binding of [$^{125}$I]GRP nor stimulates DNA synthesis in Swiss 3T3 cells. Unexpectedly, the substance P antagonist [DArg$^1$, DPro$^2$, DTrp$^{7,9}$, Leu$^{11}$] substance P (Peptide A in Table II) was found to block the secretory effects of bombesin in pancreatic acinar cells (Jensen *et al.*, 1984) and it was subsequently shown to antagonise the growth-promoting effects of bombesin in Swiss 3T3 cells (Zachary & Rozengurt, 1985*a*). It has now been shown to block all the early events leading to bombesin-stimulated mitogenesis in these cells (Table III). It also inhibits mitogenesis stimulated by vasopressin (Corps *et al.*, 1985; Zachary & Rozengurt, 1986).

SCLC cell lines grown in serum-free medium achieve 10-fold increase in number in about 12 days. Figure 3 (left) shows that the growth of H69 cells was suppressed by peptide A at 150 μM (a concentration that reversibly inhibits GRP-induced mitogenesis in Swiss 3T3 cells) but restored by

**Table II** Amino acid sequences of bombesin, substance P and the substance P antagonists tested as inhibitors of GRP-stimulated DNA synthesis

| *Bombesin* | | *pGlu* | *Gln* | *Arg* | *Leu* | *Gly* | *Asn* | *Gln* | *Trp* | *Ala* | *Val* | *Gly* | *His* | *Leu* | *Met* | *$NH_2$* |
|---|---|---|---|---|---|---|---|---|---|---|---|---|---|---|---|---|
| | | | | | *1* | *2* | *3* | *4* | *5* | *6* | *7* | *8* | *9* | *10* | *11* | |
| :tance P | | | | | Arg | Pro | Lys | Pro | Gln | Gln | Phe | Phe | Gly | Leu | Met | $NH_2$ |
| ıgonist | A | | | | DArg | Dpro | Lys | Pro | Gln | Gln | DTrp | Phe | DTrp | Leu | Leu | $NH_2$ |
| | B | | | | DArg | Pro | Lys | Pro | Gln | Gln | DTrp | Phe | DTrp | Leu | Leu | $NH_2$ |
| | C | | | | Arg | DPro | Lys | Pro | Gln | Gln | DPhe | Phe | DTrp | Leu | Met | $NH_2$ |
| | D | | | | DArg | Pro | Lys | Pro | DPhe | Gln | DTrp | Phe | DTrp | Leu | Leu | $NH_2$ |
| | E | | | | Arg | DPro | Lys | Pro | Gln | Gln | DTrp | Phe | DTrp | Leu | Met | $NH_2$ |
| | F | | | | | | | DPro | Gln | Gln | DTrp | Phe | DTrp | DTrp | Met | $NH_2$ |
| | G | | | | | | | | | Arg | DTrp | MePhe | DTrp | Leu | Met | $NH_2$ |
| | H | | | | DArg | DPro | Lys | Pro | Gln | Gln | DPhe | Phe | DHis | Leu | Met | $NH_2$ |
| | J | | | | | | | HArg | Gly | Gln | DTrp | Phe | Gly | Asp | $(OtBu)_2$ | |
| | K | | | | | | | DPro | Gln | Gln | DTrp | Phe | DTrp | Leu | Met | $NH_2$ |

**Table III** Effects of [DArg[1], DPro[2], DTrp[7,9], Leu[11]] substance P in Swiss 3T3 cells

| [DArg[1], DPro[2], DTrp[7,9], Leu[11]] substance P reversibly blocks: | *Reference* |
|---|---|
| [$^{125}$I] GRP binding | 1 |
| Cross-linking of [$^{125}$I] GRP to the $M_r$ 75,000–85,000 glycoprotein | 2 |
| 80K phosphorylation | 3 |
| EGF transmodulation | 3 |
| $Ca^{2+}$ mobilization | 4 |
| Activation of $Na^+/K^+$ pump | 4 |
| Increase in c-*fos* and c-*myc* expression | 5 |
| Stimulation of DNA synthesis by bombesin-like peptides | 1 |
| Stimulation of DNA synthesis by vasopressin | 6,7 |
| DNA synthesis stimulated by PDGF, EGF, phorbol esters and c-AMP-increasing agents is not affected | 1 |

References: 1, Zachary & Rozengurt, 1985*a*; 2, Zachary & Rozengurt, 1987*a*; 3, Zachary *et al.*, 1986; 4, Mendoza *et al.*, 1986; 5, Rozengurt & Sinnett-Smith, 1987; 6, Zachary & Rozengurt, 1986; 7, Corps *et al.*, 1985.

washing the cells and re-suspending them in serum-free medium. Figure 3 (right) demonstrates that the effect of the antagonist is concentration dependent. These results suggest a specific, non-toxic effect.

### *[DArg[1], DPhe[5], DTrp[7,9], Leu[11]] substance P*

In order to identify more potent bombesin antagonists we have tested ten substance P antagonists at 50 μM (Table II) for their ability to inhibit GRP-stimulated mitogenesis in Swiss 3T3 cells. [DArg[1], DPhe[5], DTrp[7,9], Leu[11]] substance P (Peptide D in Table II) was clearly the most potent GRP antagonist. None of the other peptides was superior to peptide A but peptide D was consistently 5-fold more potent. None of these peptides exhibited agonist activity. Clearly the substitution of DPhe for Gln at position 5 is critical to the enhanced activity of peptide D.

Detailed studies of peptide D have now been completed (Woll & Rozengurt, 1988). Its effects on the dose-response curve for GRP in an assay of DNA synthesis is shown in Figure 4 (left). The dose-response curve is shifted to the right but retains its shape, indicating that the effect of the antagonist is competitive and reversible. The dose-response curve for peptide D in the presence of GRP 3.6 nM is shown in figure 4 (right). Half-maximal inhibition of DNA synthesis was obtained with 22 μM antagonist.

**Figure 3, Left:** [DArg[1], DPro[2], DTrp[7,9], Leu[11]] substance P (peptide A in **Table II**) reversibly inhibits the growth of the H69 SCLC cell line. Cells were incubated in HITES medium (Carney *et al.*, 1981) supplemented with 0.25% bovine serum albumin in the absence (●) or presence (○) of peptide A 150 μM. Additional samples (□) were preincubated with 150 μM peptide A and resuspended in medium without antagonist. All samples were plated on day 0 with an inoculum of $5 \times 10^4$ cells. Cell number was determined using a Coulter counter. Each point represents the mean of 3 determinations. **Right:** Peptide A inhibits the growth of H69 cells in a concentration-dependent manner. Cells inoculated at $5 \times 10^4 ml^{-1}$ were incubated in serum-free medium (as above) containing varying concentrations of peptide A. Cells were counted on day 13. Each point represents the mean (± s.d.) of 5 determinations.

**Figure 4, Left:** Stimulation of DNA synthesis by GRP in quiescent Swiss 3T3 cells in the absence (■) or presence (△) of peptide D 40 μM. Cells were incubated in Dulbecco's modified Eagle's/Waymouth medium (DMEWM) containing [$^3$H] thymidine 1 μCi ml$^{-1}$ and insulin 1 μg ml$^{-1}$. DNA synthesis was assessed after 40 h by [$^3$H]thymidine incorporation into acid-insoluble material (Dicker & Rozengurt, 1980; Rozengurt & Sinnett-Smith, 1983). **Right:** Effects of varying concentrations of peptide D on DNA synthesis in the presence of 3.6 nM GRP and insulin 1 μg ml$^{-1}$.

**Figure 5, Left:** Stimulation of DNA synthesis by vasopressin in quiescent Swiss 3T3 cells in the absence (■) or presence (△) of peptide D 20 μM. Cells were incubated in DMEWM with [$^3$H] thymidine 1 μCi ml$^{-1}$ and insulin 1 μg ml$^{-1}$. DNA synthesis was assessed after 40 h by [$^3$H] thymidine incorporation into acid insoluble material (as in **Figure 4**). **Right:** Effects of varying concentrations of peptide D on DNA synthesis in the presence of 14 nM vasopressin and insulin 1 μg ml$^{-}$1.

Like peptide A, peptide D exhibits specificity in blocking mitogenesis. It does not interfere with DNA synthesis stimulated by $PBt_2$, cholera toxin with isobutylmethylxanthine, EGF or PDGF. Vasopressin-stimulated DNA synthesis, however, is markedly inhibited in a competitive and reversible manner (Figure 5). Thus peptide D is an antagonist for at least three distinct neuropeptides whose effects are mediated through specific receptors.

Binding of [$^{125}$I]GRP to the bombesin/GRP receptor in Swiss 3T3 cells is not inhibited by vasopressin or substance P. In contrast, peptide D inhibits [$^{125}$I]GRP binding in a dose-dependent manner (Figure 6, left) which is half-

Figure 6, Left: Inhibition of [$^{125}$I] GRP binding to Swiss 3T3 cells by peptide D. Quiescent cells were incubated with 1 nM [$^{125}$I] GRP and varying amounts of peptide D for 30 min (Zachary) & Rozengurt, 1985*a*). Binding is expressed as percentage of the specific binding obtained in the absence of antagonist. **Right:** Effects of peptide D on affinity labelling of bombesin receptor-associated $M_r$ 75,000–85,000 protein. Confluent Swiss 3T3 cells were incubated for 2 h in binding medium containing 0.5 nM [$^{125}$I] GRP and varying amounts of peptide D. After washing, they were incubated for 15 min with the cross-linking agent ethylene glycol bis (succinimidyl succinate) then solubilized and electrophoresed on a 10% polyacrylamide gel (inset) (Zachary & Rozengurt, 1987*a*). Results are expressed as percentage of the control on scanning densitometry of the autoradiographs.

**Figure 7** Effects of peptide D on proliferation of SCLC and NSCLC cell lines. Cells were incubated in HITES medium supplemented with 0.25% albumin and 1% fetal bovine serum in the absence (□) or presence (■) of peptide D 40μM. Cell number was determined when maximal growth had been achieved, at Day 11 for H128 and at Day 7 for sk-mes-1 and sk-lu-1. Results are expressed as the mean (±s.d.) of 5 determinations.

maximal at 2.3 μM. Cross-linking of the $M_r$ 75,000–85,000 protein component of the bombesin/GRP receptor is inhibited by peptide D with half-maximal effect at 5.5 μM (Figure 6, right). These findings indicate that the effects of peptide D on mitogenesis stimulated by the bombesins are mediated through the specific bombesin receptor.

Peptide D inhibits the growth of SCLC cell lines *in vitro* with half-maximal effect at 24 μM in the H69 cell line (Woll & Rozengurt, 1988). As in the Swiss 3T3 system, the new antagonist is ~5-fold more potent than peptide A. The effects on non-small cell lung cancer (NSCLC) cell lines are less striking. Figure 7 contrasts the effects of the new antagonist at 40μM on growth in chemically defined medium supplemented with 1% serum of the H128 SCLC line and two NSCLC lines, sk-mes-1 (squamous) and sk-lu-1 (adenocarcinoma). Although the NSCLC lines do not grow as readily as SCLC line *in vitro*, the antagonist has much less inhibitory effect on them than on the SCLC line.

### Conclusion

The use of the Swiss 3T3 murine fibroblast system in chemically defined conditions has allowed comprehensive investigation into the mechanism of action of bombesins as mitogens. Detailed knowledge of these growth-regulatory pathways has led to formulation of hypotheses concerning the control of tumour growth in SCLC. These hypotheses will be subject to critical testing using synthetic bombesin antagonists such as [DArg$^1$, DPhe$^5$, DTrp$^{7,9}$, Leu$^{11}$] substance P. Although this antagonist profoundly inhibits the growth of SCLC *in vitro*, it is not yet clear whether this effect is mediated through bombesin alone or other neuropeptide growth factors. Both [DArg$^1$, DPro$^2$, DTrp$^{7,9}$, Leu$^{11}$] substance P and [DArg$^1$, DPhe$^5$, DTrp$^{7,9}$, Leu$^{11}$] substance P antagonise the effects of three neuropeptides, bombesin, vasopressin and substance P through their distinct receptors. It is tempting to speculate that these antagonists could bind to a common domain in the three receptors. A possible model for this is provided by the recent molecular cloning of a receptor for the neuropeptide substance K, which has seven transmembrane segments and shows amino acid sequence homology with other neurotransmitter receptors and with the *mas* human oncogene (Hanley & Jackson, 1987; Masu *et al.*, 1987). More potent and specific bombesin antagonists could prove useful as investigative tools and potential therapeutic agents with high tissue penetration.

### References

ANASTASI, A., ERSPAMER, V. & BUCCI, M. (1971). Isolation and structure of bombesin and alytesin, two analogous active peptides from the skin of the European amphibians *Bombina* and *Alytes*. *Experientia.*, **27**, 166.

BAILAR, J.C. & SMITH, E.M. (1986). Progress against cancer? *N. Engl. J. Med.*, **314**, 1226.

BALLESTER, R. & ROSEN, O.M. (1985). Fate of immunoprecipitable protein kinase C in $GH_3$ cells treated with phorbol 12-myristate 13-acetate. *J. Biol. Chem.*, **260**, 15194.

BERGERON, J.J.M., CRUZ, J., KHAN, M.N. & POSNER, B.I. (1985). Uptake of insulin and other ligands into receptor-rich endocytic components of target cells: the endosomal apparatus. *Ann. Rev. Physiol.*, **47**, 383.

BERRIDGE, M.J. & IRVINE, R.F. (1984). Inositol trisphosphate, a novel second messenger in cellular signal transduction. *Nature*, **312**, 315.

BISHOP, R., MARTINEZ, R., WEBER, M.J. & 4 others (1985). Protein phosphorylation in a tetradecanoyl phorbol acetate-non proliferative variant of 3T3 cells. *Mol. Cell. Biol.*, **5**, 2231.

BLACKSHEAR, P.J., WEN, L., GLYNN, B.P. & WITTERS, L.A. (1986). Protein kinase C-stimulated phosphorylation *in vitro* of a $M_r$ 80,000 protein phosphorylated in response to phorbol esters and growth factors in intact fibroblasts. *J. Biol. Chem.*, **261**, 1459.

BLACKSHEAR, P.J., WITTERS, L.A., GIRARD, P.R., KUO, U.F. & QUAMO, S.N. (1985). Growth-factor stimulated protein phosphorylation in 3T3-LI cells; evidence for protein kinase C-dependent and independent pathways. *J. Biol. Chem.*, **260**, 13304.

BROWN, K.D., BLAY, J., IRVINE, R.F., HESLOP, J.P. & BERRIDGE, M.J. (1984). Reduction of epidermal growth factor receptor affinity by heterologous ligands: evidence for a mechanism involving the breakdown of phosphoinositides and the activation of protein kinase C. *Biochem. Biophys. Res. Commun.*, **123**, 377.

CARNEY, D.N., BUNN, P.A., GAZDAR, A.F., PAGAN, J.A. & MINNA, J.D. (1981). Selective growth in serum-free hormone-supplemented medium of tumor cells obtained by biopsy from patients with small cell carcinoma of the lung. *Proc. Natl Acad. Sci. USA.*, **78**, 3185.

CARNEY, D.N., CUTTITTA, F., MOODY, T.W. & MINNA, J.D. (1987). Selective stimulation of small cell lung cancer clonal growth by bombesin and gastrin-releasing peptide. *Cancer Res.*, **47**, 821.

CIRILLO, D.M., GAUDINO, G., NALDINI, L. & COMOGLIO, P.M. (1986). Receptor for bombesin with associated tyrosine kinase activity. *Mol. Cell. Biol.*, **6**, 4641.

CORPS, A.N., REES, L.H. & BROWN, K.D. (1985). A peptide that inhibits the mitogenic stimulation of Swiss 3T3 cells by bombesin or vasopressin. *Biochem. J.*, **231**, 781.

CUTTITTA, F., CARNEY, D.N., MULSHINE, J. & 4 others (1985). Bombesin-like peptides can function as autocrine growth factors in human small-cell lung cancer. *Nature*, **316**, 823.

DICKER, P. & ROZENGURT, E. (1980). Phorbol esters and vasopressin stimulate DNA synthesis by a common mechanism. *Nature*, **287**, 607.

ERISMAN, M.D., LINNOILA, R.I., HERNANDEZ, O., DIAUGUSTINE, R.P. & LAZARUS, L.H. (1982). Human lung small-cell carcinoma contains bombesin. *Proc. Natl Acad. Sci. USA*, **79**, 2379.

EVERED, D., NUGENT, J. & WHELAN, J. (eds) (1985). *Growth Factors in Biology and Medicine*. Ciba Foundation Symposium 116, Pitman: London.

GHATEI, M.A., SHEPPARD, M.N., HENZEN-LOGMAN, S., BLANK, M.A., POLAK, J.M. & BLOOM, S.R. (1983). Bombesin and vasoactive intestinal polypeptide in the developing lung: marked changes in acute respiratory distress syndrome. *J. Clin. Endocrinol. Metab.*, **57**, 1226.

GOLDSTEIN, J.L., BROWN, M.S., ANDERSON, R.G.W., RUSSELL, D.W. & SCHNEIDER, W.J. (1985). Receptor-mediated endocytosis. *Ann. Rev. Cell Biol.*, **1**, 1.

HANLEY, M.R. & JACKSON, T. (1987). Return of the magnificent seven. *Nature*, **329**, 766.

HELDIN, C.-H. & WESTERMARK, B. (1984). Growth factors: mechanism of action and relation to oncogenes. *Cell*, **37**, 9.

HESLOP, J.P., BLAKELEY, D.M., BROWN, K.D., IRVINE, R.F. & BERRIDGE, M.J. (1986). Effects of bombesin and insulin on inositol(1,4,5) trisphosphate and inositol(1,3,4) trisphosphate formation in Swiss 3T3 cells. *Cell*, **47**, 703.

ISACKE, C.M., MEISENHELDER, J., BROWN, K.D., GOULD, K.L., GOULD, S.J. & HUNTER, T. (1986). Early phosphorylation events following the treatment of Swiss 3T3 cells with bombesin and the mammalian bombesin-related peptide, gastrin-releasing peptide. *EMBO J.*, **5**, 2889.

JAMES, R. & BRADSHAW, R.A. (1984). Polypeptide growth factors. *Ann. Rev. Biochem.*, **53**, 259.

JENSEN, R.T., JONES, S.W., FOLKERS, K. & GARDNER, J.D. (1984). A synthetic peptide that is a bombesin receptor antagonist. *Nature*, **309**, 61.

KING, A.C. & CUATRECASAS, P. (1981). Peptide hormone-induced receptor mobility, aggregation and internalization. *N. Engl. J. Med.*, **305**, 77.

LEBACQ-VERHEYDEN, A.M., KRYSTAL, G., MARKOWITZ, S., WAY, J., SAUSVILLE, E. & BATTEY, J. (1987). Molecular analysis of the expression of mammalian prepro-gastrin releasing peptide genes. *Regul. Peptides*, **19**, 122.

LETTERIO, J.J., COUGHLIN, S.R. & WILLIAMS, L.T. (1986). Pertussis toxin-sensitive pathway in the stimulation of c-*myc* expression and DNA synthesis by bombesin. *Science*, **234**, 1117.

LEZOCHE, E., BASSO, N. & SPERANZA, V. (1981). Actions of bombesin in man. In *Gut hormones*, Bloom, S.R. & Polak, J.M. (eds) p. 419. Churchill Livingstone: London.

LIN, C.R., CHEN, W.S., LAZAR, C.S. & 4 others (1986). Protein kinase C phosphorylation at Thr 654 of the unoccupied EGF receptor and EGF binding regulate functional receptor loss by independent mechanisms. *Cell*, **44**, 839.

LOPEZ-RIVAS, A., MENDOZA, S.A., NÅNBERG, E., SINNETT-SMITH, J. & ROZENGURT, E. (1987). The $Ca^{2+}$-mobilizing actions of platelet-derived growth factor differ from those of bombesin and vasopressin in Swiss 3T3 cells. *Proc. Natl Acad. Sci., USA*, **84**, 5768.

MASU, Y., NAKAYAMA, K., TAMAKI, H., HARADA, Y., KUNO, M. & NAKANISHI, S. (1987). cDNA cloning of bovine substance-K receptor through oocyte expression system. *Nature*, 329, 836.

McDONALD, T.J., JORNVALL, H., NILSSON, G. & 4 others (1979). Characterization of a gastrin releasing peptide from porcine non-antral gastric tissue. *Biochem. Biophys. Res. Commun.*, **90**, 227.

MENDOZA, S.M., SCHNEIDER, J.A., LOPEZ-RIVAS, A., SINNETT-SMITH, J.W. & ROZENGURT, E. (1986). Early events elicited by bombesin and structurally related peptides in quiescent Swiss 3T3 cells. II. Changes in $Na^+$ and $Ca^{2+}$ fluxes, $Na^+/K^+$ pump activity, and intracellular pH. *J. Cell Biol.*, **102**, 2223.

MINAMINO, N., KANGAWA, K. & MATSUO, H. (1983). Neuromedin B: a novel bombesin-like peptide identified in porcine spinal cord. *Biochem. Biophys. Res. Commun.*, **114**, 541.

MINAMINO, N., KANGAWA, K. & MATSUO, H. (1984). Neuromedin C: a bombesin-like peptide identified in porcine spinal cord. *Biochem. Biophys. Res. Commun.*, **119**, 14.

MINAMINO, N., SUDOH, T., KANGAWA, K. & MATSUO, H. (1985). Neuromedin B-32 and B-30: two 'big' neuromedin B identified in porcine brain and spinal cord. *Biochem. Biophys. Res. Commun.*, **130**, 685.

MOODY, T.W., PERT, C.B., GAZDAR, A.F., CARNEY, D.N. & MINNA, J.D. (1981). High levels of intracellular bombesin characterize human small cell lung carcinoma. *Science*, **214**, 1246.

MUIR, J.G. & MURRAY, A.W. (1987). Bombesin and phorbol ester stimulate phosphatidylcholine hydrolysis by phospholipase C: evidence for a role of protein kinase C. *J. Cell. Physiol.*, **130**, 382.

NISHIZUKA, Y. (1984). The role of protein kinase C in cell surface signal transduction and tumour promotion. *Nature*, **308**, 693.

PALUMBO, A.P., ROSSINO, P. & COMMOGLIO, P.M. (1986). Bombesin stimulation of c-*fos* and c-*myc* gene expression in cultures of Swiss 3T3 cells. *Exp. Cell Res.*, **167**, 276.

PRICE, J., PENMAN, E., BOURNE, G.L. & REES, L.H. (1983). Characterisation of bombesin-like immunoreactivity in human fetal lung. *Regul. Peptides*, **7**, 315.

RODRIGUEZ-PENA, A. & ROZENGURT, E. (1984). Disappearance of $Ca^{2+}$-sensitive, phospholipid-dependent protein kinase activity in phorbol ester-treated 3T3 cells. *Biochem. Biophys. Res. Commun.*, **120**, 1053.

RODRIGUEZ-PENA, A. & ROZENGURT, E. (1985). Serum, like phorbol esters, rapidly activates protein kinase C in intact quiescent fibroblasts. *EMBO J.* **4**, 71.

RODRIGUEZ-PENA, A. & ROZENGURT, E. (1986). Phosphorylation of an acidic molecular weight 80,000 cellular protein in a cell-free system and intact Swiss 3T3 cells: a specific marker of protein kinase C activity. *EMBO J.*, **5**, 77.

ROSENBLATT, M. (1986). Peptide hormone antagonists that are effective *in vivo*. Lessons from parathyroid hormone. *N. Engl. J. Med.*, **315**, 1004.

ROZENGURT, E. (1983). Growth factors, cell proliferation and cancer: an overview. *Mol. Biol. Med.*, **1**, 169.

ROZENGURT, E. (1986). Early signals in the mitogenic response. *Science*, **234**, 161.

ROZENGURT, E., MURRAY, M., ZACHARY, I. & COLLINS, M. (1987). Protein kinase C activation enhances cAMP accumulation in Swiss 3T3 cells: inhibition by pertussis toxin. *Proc. Natl Acad. Sci. USA*, **84**, 2282.

ROZENGURT, E., RODRIGUEZ-PENA, A. & SMITH, K.A. (1983). Phorbol esters, phospholipase C, and growth factors rapidly stimulate the phosphorylation of a $M_r$ 80,000 protein in intact quiescent 3T3 cells. *Proc. Natl Acad. Sci., USA*, **80**, 7244.

ROZENGURT, E., RODRIGUEZ-PENA, A., COOMBS, M. & SINNETT-SMITH, J. (1984). Diacylglycerol stimulates DNA synthesis and cell division in mouse 3T3 cells: role of $Ca^{2+}$-sensitive phospholipid-dependent protein kinase C. *Proc. Natl Acad. Sci., USA*, **81**, 5748.

ROZENGURT, E. & SINNETT-SMITH, J. (1983). Bombesin stimulation of DNA synthesis and cell division in cultures of Swiss 3T3 cells. *Proc. Natl Acad. Sci., USA*, **80**, 2936.

ROZENGURT, E. & SINNETT-SMITH, J.W. (1987). Bombesin induction of c-*fos* and c-*myc* proto-oncogenes in Swiss 3T3 cells: significance for the mitogenic response. *J. Cell. Physiol.*, **131**, 218.

SAUSVILLE, E.A., LEBACQ-VERHEYDEN, A.-M., SPINDEL, E.R., CUTTITTA, F., GAZDAR, A.F. & BATTEY, J.F. (1986). Expression of the gastrin-releasing peptide gene in human small cell lung cancer: Evidence for alternative processing resulting in three distinct mRNAs. *J. Biol. Chem.*, **261**, 2451.

SPINDEL, E.R., CHIN, W.W., PRICE, J., REES, L.H., BESSER, G.M. & HABENER, J.F. (1984). Cloning and characterization of cDNAs encoding human gastrin-releasing peptide. *Proc. Natl Acad. Sci., USA*, **81**, 5699.

SPINDEL, E.R., SUNDAY, M.E., HOFLER, H., WOLFE, H.J., HABENER, J.F. & CHIN, W.W. (1987). Transient elevation of messenger RNA encoding gastrin-releasing peptide, a putative pulmonary growth factor in human fetal lung. *J. Clin. Invest.*, **80**, 1172.

SPIRO, S.G. (1985). Chemotherapy of small cell lung cancer. *Clinics Oncol.*, **4**, 105.

SPORN, M.B. & ROBERTS, A.B. (1986). Peptide growth factors and inflammation, tissue repair, and cancer. *J. Clin. Invest.*, **78**, 329.

STABEL, S., RODRIGUEZ-PENA, A., YOUNG, S., ROZENGURT, E. & PARKER, P.J. (1987). Quantitation of protein kinase C by immunoblot-expression in different cell lines and response to phorbol esters. *J. Cell. Physiol.*, **130**, 111.

SUZUKI, M. YAMAGUCHI, K., ABE, K. & 9 others (1987). Detection of gastrin-releasing peptide mRNA in small cell lung carcinomas and medullary thyroid carcinomas using synthetic oligodeoxyribonucleotide probes. *Jpn J. Clin. Oncol.*, **17**, 157.

TAKUWA, N., TAKUWA, Y., BOLLAG, W.E. & RASMUSSEN, H. (1987). The effects of bombesin on polyphosphoinositide and calcium metabolism in Swiss 3T3 cells. *J. Biol. Chem.*, **262**, 182.

VARA, F., SCHNEIDER, J.A. & ROZENGURT, E. (1985). Ionic responses rapidly elicited by activation of protein kinase C in quiescent Swiss 3T3 cells. *Proc. Natl Acad. Sci., USA*, **82**, 2384.

WAKSHULL, E.M. & WHARTON, W. (1985). Stabilized complexes of epidermal growth factor and its receptor on the cell surface stimulate RNA synthesis but not mitogenesis. *Proc. Natl Acad. Sci., USA*, **82**, 8513.

WEBER, S. ZUCKERMAN, J.E., BOSTWICK, D.G., BENSCH, K.G., SIKIC, B.I. & RAFFIN, T.A. (1985). Gastrin releasing peptide is a selective mitogen for small cell lung carcinoma *in vitro*. *J. Clin. Invest.*, **75**, 306.

WEINSTEIN, B. (1987). Growth factors, oncogenes and multistage carcinogenesis. *J. Cell. Biochem.*, **33**, 213.

WHARTON, J., POLAK, J.M., BLOOM, S.R. & 4 others (1978). Bombesin-like immunoreactivity in the lung. *Nature*, **273**, 769.

WOLL, P.J. & ROZENGURT, E. (1988). [DArg$^1$, DPhe$^5$, DTrp$^{7,9}$, Leu$^{11}$] substance P, a potent bombesin antagonist in murine Swiss 3T3 cells, inhibits the growth of human small cell lung cancer cells *in vitro*. *Proc. Natl Acad. Sci., USA*, **85**, 1859.

WOOD, S.M., WOOD, J.R., GHATEI, M.A., LEE, Y.C. O'SHAUGHNESSY, D. & BLOOM, S.R. (1981). Bombesin, somatostatin and neurotensin-like immunoreactivity in bronchial carcinoma. *J. Clin. Endocrin. Metabol.*, **53**, 1310.

YAMAGUCHI, K., ABE, K., KAMEYA, T. & 4 others (1983). Production and molecular size heterogeneity of immunoreactive gastrin-releasing peptide in fetal and adult lungs and primary lung tumours. *Cancer Res.*, **43**, 3932.

ZACHARY, I., MILLAR, J., NÅNBERG, E., HIGGINS, T. & ROZENGURT, E. (1987*a*). Inhibition of bombesin-induced mitogenesis by pertussis toxin-dissociation from phospholipase C pathway. *Biochem. Biophys. Res. Commun.*, **146**, 456.

ZACHARY, I., & ROZENGURT, E. (1985*a*). High-affinity receptors for peptides of the bombesin family in Swiss 3T3 cells. *Proc. Natl Acad. Sci. USA.*, **82**, 7616.

ZACHARY, I. & ROZENGURT, E. (1985*b*). Modulation of the epidermal growth factor receptor by mitogenic ligands: effects of bombesin and role of protein kinase C. *Cancer Surveys*, **4**, 729.

ZACHARY, I. & ROZENGURT, E. (1986). A substance P antagonist also inhibits specific binding and mitogenic effects of vasopressin and bombesin-related peptides in Swiss 3T3 cells. *Biochem. Biophys. Res. Commun.*, **137**, 135.

ZACHARY, I. & ROZENGURT, E. (1987*a*). Identification of a receptor for peptides of the bombesin family in Swiss 3T3 cells by affinity cross-linking. *J. Biol. Chem.*, **262**, 3947.

ZACHARY, I. & ROZENGURT, E. (1987*b*). Internalization and degradation of peptides of the bombesin family in Swiss 3T3 cells occurs without ligand-induced receptor down-regulation. *EMBO J.*, **6**, 2233.

ZACHARY, I., SINNETT-SMITH, J.W. & ROZENGURT, E. (1986). Early events elicited by bombesin and structurally related peptides in quiescent Swiss 3T3 cells. I. Activation of protein kinase C and inhibition of epidermal growth factor binding. *J. Cell Biol.*, **102**, 2211.

ZACHARY, I., WOLL, P.J. & ROZENGURT, E. (1987*b*). A role for neuropeptides in the control of cell proliferation. *Devel. Biol.*, **124**, 295.